# Clinical Feasibility of 5.0 T MRI/MRCP in Characterizing Pancreatic Cystic Lesions: Comparison with 3.0 T and MDCT

**DOI:** 10.3390/diagnostics14212457

**Published:** 2024-11-02

**Authors:** Huijia Zhao, Qiang Xu, Ruichen Gao, Bohui Yin, Gan Sun, Ke Xue, Yuxin Yang, Enhui Li, Liang Zhu, Feng Feng, Wenming Wu

**Affiliations:** 1 Department of General Surgery, Peking Union Medical College Hospital, Chinese Academy of Medical Science and Peking Union Medical College, Beijing 100730, China; drzhaohuijia@163.com (H.Z.); xuqiang@pumch.cn (Q.X.); gaoruichen@student.pumc.edu.cn (R.G.); yinbohui@student.pumc.edu.cn (B.Y.); 2 State Key Laboratory of Complex Severe and Rare Diseases, Beijing 100730, China; ffeng@pumch.cn; 3 Theranostics and Translational Research Center, National Infrastructures for Translational Medicine, Institute of Clinical Medicine, Peking Union Medical College Hospital, Chinese Academy of Medical Sciences, Beijing 100730, China; 18911127990@163.com; 4 United Imaging Research Institute of Intelligent Imaging, Beijing 100089, China; ke.xue@cri-united-imaging.com (K.X.); yuxin.yang@cri-united-imaging.com (Y.Y.); enhui.li@cri-united-imaging.com (E.L.); 5 Department of Radiology, Peking Union Medical College Hospital, Chinese Academy of Medical Science and Peking Union Medical College, Beijing 100730, China

**Keywords:** 5.0 T MRI, pancreatic cystic lesions, image quality, diagnostic accuracy

## Abstract

**Objectives:** To assess the feasibility of 5.0 T magnetic resonance imaging (MRI) in characterizing pancreatic cystic lesions (PCLs), compared with 3.0 T MRI and multidetector computed tomography (MDCT). **Methods:** Thirty-five patients with PCLs underwent 5.0 T MR alongside 3.0 T MR or MDCT. Two observers measured subjective and objective image quality scores. The consistency of two observers between 5.0 T and 3.0 T was calculated by intraclass correlation coefficients. The characteristics of PCLs and their specific diagnosis, as well as benignity/malignancy, were evaluated across MDCT, 3.0 T, and 5.0 T MRI. **Results:** The 5.0 T MR demonstrated significantly higher subjective image quality and SNR on T1WI compared to that in 3.0 T MR (*p* < 0.05). The 5.0 T MRI identified more cyst lesions than the 3.0 T MRI (40 and 32) and MDCT (82 and 56). The sensitivity, specificity, and accuracy for differentiating benign from malignant lesions with 5.0 T MRI (75%, 100%, and 91.4%, respectively) surpassed those of 3.0 T MRI and MDCT. The accuracy of the specific diagnosis of PCLs at 5.0 T MRI (80%) was superior to 3.0 T MRI and MDCT. **Conclusions:** 5.0 T MRI exhibits certain superiority in delineating details of PCLs and in clinical diagnostic accuracy, outperforming MDCT and 3.0 T MRI while maintaining sufficient image quality.

## 1. Introduction

Pancreatic cystic lesions (PCLs) represent a heterogeneous group of lesions with varying histological profiles, encompassing non-neoplastic lesions, benign neoplastic lesions, neoplasms with malignant potential, and frank malignancies [1]. The incidental detection of PCLs has risen concomitantly with advancements in multidetector computed tomography (MDCT) and magnetic resonance imaging (MRI) [2]. The nature and biological behavior of different types of PCLs vary greatly, some of which may present with or progress to malignancy. Thus, accurate diagnosis of these cystic lesions is crucial for risk stratification and clinical management [3].

MRI is pivotal in the evaluation of patients with PCLs. Its applications are extensive, ranging from screening high-risk individuals for pancreatic cancer to evaluating pancreatic cysts and ambiguous pancreatic lesions. Although MDCT is widely available and has higher spatial resolution compared to MRI, MRI is known for superior soft tissue contrast and allows for multi-sequence imaging in different planes, potentially leading to better differentiation of lesions [4]. Despite its significance, pancreatic MRI faces challenges due to the small size of the pancreas, its upper abdominal location adjacent to the intestine, and its positioning beneath the diaphragm. In recent years, the field of ultra-high field MRI has progressed rapidly [5,6,7]. Nevertheless, the use of ultra-high field MRI for human imaging is currently capped at 7.0T, with higher strengths being relegated to preclinical research, which is only confined to imaging the head and limb joints, with abdominal imaging remaining unfeasible [8]. The development of a 5.0 T whole-body MRI scanner represents a milestone as it is the first to facilitate ultra-high field MRI for the upper abdomen [9]. Initial studies involving a small cohort of healthy volunteers have indicated its potential in pancreatic imaging, yielding results on par with the conventional 3.0 T MRI [10]. Despite these advancements, research into the diagnostic capabilities of 5T MRI for pancreatic lesions remains unexplored.

Hence, the purpose of our study is to evaluate the diagnostic performance of 5.0 T MRI in PCLs in comparison with 3.0 T MRI and MDCT.

## 2. Patients and Methods

### 2.1. Patients

The prospective study was approved by our ethics committee (the Ethics Committee of Peking Union Medical College Hospital), and all patients signed informed consent. From October 2021 to March 2023, we prospectively enrolled patients with suspected PCLs based on previous imaging studies for 5.0 T MRI/MRCP studies. PCLs were characterized as any pancreatic lesions with a cystic appearance at abdominal ultrasound, CT, or MRI.

The exclusion criteria included patients who did not have MDCT or 3.0 T MR at our institution within one year of the 5.0 T MR study and those lacking a definite pathological diagnosis or with follow-up periods under one year. Consistent with prior research [11], lesions such as intraductal oncocytic papillary neoplasm, pancreatic neuroendocrine tumor above G1, solid pseudopapillary neoplasm, mucinous cystic neoplasm, and high-grade dysplasia/invasive cancer of intraductal papillary mucinous neoplasm (IPMN) were categorized as malignant lesions. Patients who had a pathological diagnosis other than the above-mentioned pathological types or patients who did not exhibit worrisome or high-risk features and were followed up without progression are defined as having benign PCLs [12].

### 2.2. 5.0 T MRI Protocol

All patients underwent 5.0 T pancreatic MRI on a prototype whole-body MR scanner (uMR Jupiter, United Imaging Healthcare, Shanghai, China) with a 24-channel phased-array surface receive coil. An 8-channel parallel volumetric transmit coil was used to mitigate dielectric artifacts through independent channel control. During the prescan calibration, single-channel B1 maps are acquired for each of the 8 channels to determine individual sensitivity maps. Using these B1 field distributions, the optimal radio frequency (RF) homogenization coefficients are computed and subsequently applied to the spectrometer for subsequent MRI scans. With the following formulation, the RF field can be designed by solving the following optimization problem based on magnitude least squares (MLS) [13], where matrix A represents the sensitivity maps obtained from a prescan for each transmit channel. b denotes the complex weighting of each channel and T represents the target flip angle. R(b) denotes a regularization term to control integrated and peak RF power. The sequences included axial and coronal T2-weighted fast spin-echo (FSE) sequences with fat saturation and respiratory trigger, 3D T1-weighted spoiled gradient-echo (GRE) sequence, diffusion-weighted imaging (DWI) with reduced FOV and respiratory trigger and 3D magnetic resonance cholangiopancreatography (MRCP) based on a single FSE sequence. Detailed parameters of each sequence are provided in Table 1. The contrast-enhanced sequence was not performed.

### 2.3. Electromagnetic Simulations and Validation of B1 Filed

Simulations were conducted using a Sim4life dataset comprising 15 human models [14]. It focused on a 5mm thick transverse section at the pancreas’s center for single-layer RF shimming, facilitating thorough parameter analysis. To ensure comparability with real system calibration, we standardized the average magnetic field within the upper abdominal region across all simulations. Additionally, the transmit B1 maps of pancreatic are estimated using the saturated TurboFLASH method [15] from a healthy male volunteer scanned on the 5.0 T MRI system to validate the effectiveness of the above RF shimming strategy. The coefficient of variation (CV) was employed to assess the uniformity of the transmit field.

### 2.4. 3.0 T MRI Protocol

Twenty-one of those patients also underwent pancreatic MRI on various 3.0 T MR systems (MAGNETOM Skyra, Siemens Healthineers, Erlangen, Germany; GE Discovery MR 750, GE Healthcare, Waukesha, WI, USA and Ingenia CX, Philips Medical Systems, Best, The Netherlands). The protocols included the following sequences or their analogs, which were used in the clinical routine at our institution: volume-interpolated breath-hold T1-weighted imaging (T1WI), turbo-spin-echo fat-saturated T2-weighted imaging (T2WI), axial and coronal half-Fourier acquisition single-shot turbo-spin-echo T2WI, and DWI with b values of 0 and 800 mm^2^/s, and isotropic 3D MRCP. Detailed parameters of different 3.0 T MRIs are summarized in Table 1. A multi-phase contrast-enhanced sequence was performed but not analyzed in this study.

### 2.5. MDCT Protocol

Thirty-three patients also underwent multi-phase contrast-enhanced CT at 64-row or 128-row MDCT scanners (Somatom Definition Flash and Force, Siemens Healthinners, Forchheim, Germany, and GE Discovery CT750 HD, GE Healthcare, Waukesha, WI, USA). Scanning parameters were slightly different among different vendors, but all included non-enhanced, pancreatic arterial phase, and portal venous phase images. After the non-enhanced scan, nonionic iodinated contrast material (370 mg I/mL) was injected intravenously at a rate of 3 mL/s, with 1.5 mL/kg of body weight. An automatic bolus tracking technique was utilized, and a pancreatic arterial phase scan was initiated 15 s after aortic enhancement of 100 HU, followed by a portal venous phase with a 30 s delay after the initiation of the previous phase scanning. Images were reconstructed at 1 mm section thickness. Multiplanar projection reformation in the coronal plane and 2D curved planar reformation along the main pancreatic duct were routinely obtained.

### 2.6. Image Analysis

Two radiologists, with 5 and 12 years of experience in abdominal imaging, respectively, independently reviewed all 5.0 T MRI, 3.0 T MRI, and MDCT images. Both radiologists were blinded to the patient’s identities, clinical and laboratory findings, and histologic diagnosis. Moreover, for 5.0 T and 3.0 T MRI, the radiologists were blinded to the field strength. To further minimize recall bias, the reviews of 5.0 T MRI, 3.0 T MRI, and MDCT were spaced at least two weeks apart. During each session, the radiologists recorded the following observations for comparison: the location, size, and number of cystic lesions, the diameter of the main pancreatic duct (MPD) and the extent of MPD widening (>5mm), the presence or absence of cyst communication with MPD, mural nodules and intracystic septa, intracystic fluid, and peripancreatic infiltration.

Image quality for both 5.0 T MRI and 3.0 T MRI was independently evaluated by two radiologists across various sequences, including T1WI, T2WI, DWI (b = 800 s/mm^2^), and MRCP. The assessment criteria included the visibility of the pancreas regions (head, body, tail), visibility of the splenic vein, the presence of artifacts (ghosting artifacts, distortion artifacts, motion artifacts, etc.), signal homogeneity, and overall image quality. A Likert five-point scale was utilized for the subjective evaluation: 1—extremely poor image quality, non-diagnostic; 2—poor image quality; 3—fair image quality; 4—good image quality; 5—excellent image quality [10].

For the quantitative analysis, signal-to-noise ratio (SNR) and contrast–noise ratio (CNR) were calculated within the cystic lesions on T1WI and T2WI. SNR was defined as the ratio between the mean signal intensity within the lesions and the standard deviation of background noise. CNR was calculated as the absolute difference in SI between the lesion and its adjacent tissue divided by the standard deviation of background noise. The regions of interest (ROIs) were manually delineated by the two radiologists in consensus on the T1WI and T2WI, targeting the tissue of interest. Care was taken to select ROIs comprising 150 pixels while avoiding artifact-affected areas, vessels, and the pancreatic duct. The location of the ROI was kept as consistent as possible between 5.0 T and 3.0 T MRI. An example of ROI selection is shown in Supplement Figure S1.

### 2.7. Statistical Analysis

Statistical analyses were carried out with SPSS (Version 24.0; IBM Corporation, Chicago, IL, USA). Interobserver agreement was assessed with an intraclass correlation coefficient (ICC). The level of agreement was categorized as follows: ICC = 0–0.2, slight agreement; 0.21–0.4, fair agreement; 0.41–0.60, moderate agreement; 0.61–0.8, substantial agreement; and >0.8, excellent agreement [16]. Subjective image quality and quantitative analysis results at 5.0 T and 3.0 T MRI were compared with paired Wilcoxon tests, taking into account the non-parametric nature of the data. The χ^2^ test was applied to analyze categorical imaging features associated with the depiction of PCLs, allowing for the assessment of any statistically significant associations between these features across different imaging modalities. Sensitivity and specificity and areas under the receiver operating characteristics curve (AUCs) were computed among 5.0 T MRI, 3.0 T MRI, and CT to assess the malignant potential of PCLs. A *p*-value of less than 0.05 was considered statistically significant.

## 3. Results

### 3.1. Patient Characteristics

**Figure 1:**
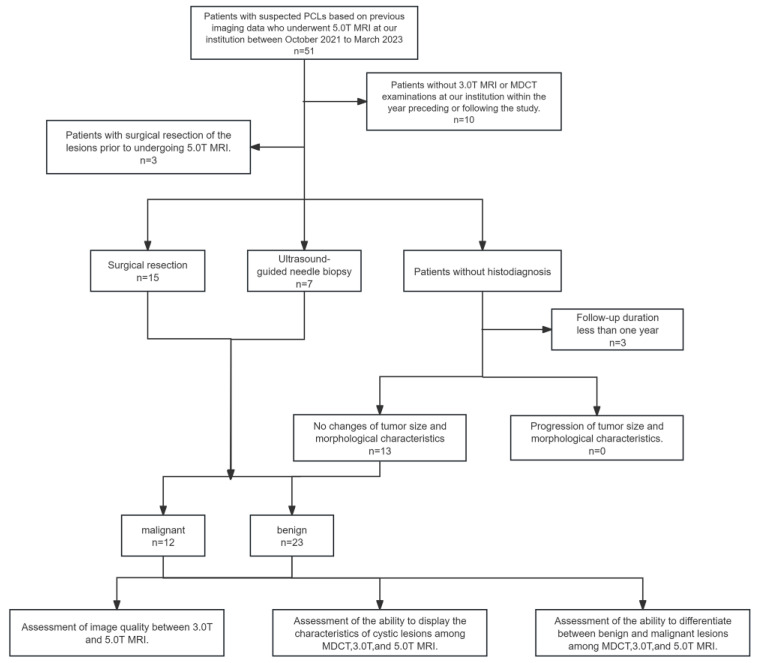
Flow diagram of enrolled patients. Of the patients included in the 5.0 T MRI study, 33 had MDCT images, and 21 had 3.0 T MRI scans. To ensure consistency in imaging, the interval between MDCT or 3.0 T MRI and 5.0 T MRI was kept under one year (mean interval: 152 days between 5.0 T MRI and MDCT, 142 days between 5.0 T MRI and 3.0 T MRI).

A total of 35 patients (12 men, average age 57 years, and range 28–87 years) were included in this study. The demographic data of the patients are summarized in Table 2. Eighteen patients were diagnosed as IPMNs, including 13 branch duct IPMNs and 5 mixed-type IPMNs. A total of 15 patients (42.86%) underwent surgery, while 20 patients opted for regular follow-up, of whom 7 (20%) had experienced ultrasound-guided biopsies to determine benignity or malignancy. According to the pathological and follow-up results, 12 patients were diagnosed with malignant PCLs and 23 patients with benign PCLs (Figure 1).

Of the patients included in the 5.0 T MRI study, 33 had MDCT images, and 21 had 3.0 T MRI scans. To ensure consistency in imaging, the interval between MDCT or 3.0 T MRI and 5.0 T MRI was kept under one year (mean interval: 152 days between 5.0 T MRI and MDCT, 142 days between 5.0 T MRI and 3.0 T MRI).

### 3.2. Image Quality and Interobserver Agreement: 5.0 T MRI vs. 3.0 T MRI

The evaluation of subjective and objective image quality metrics showed fair to excellent interobserver agreement for both 5.0 T and 3.0 T MRI (ICC = 0.596–0.955) (Table 3). Specifically, for the 5.0 T MRI, the observers achieved excellent agreement across all sequences in terms of image quality scores, SNR, and CNR, with ICC values ranging from 0.801 to 0.955. In contrast, the 3.0 T MRI assessments showed moderate agreement for the image quality score of T1WI and T2WI with ICCs of 0.596 and 0.603, respectively. Substantial agreement on the SNR of T2WI (ICC = 0.788) and CNR of both T1WI and T2WI (ICC = 0.792) was obtained.

**Figure 2:**
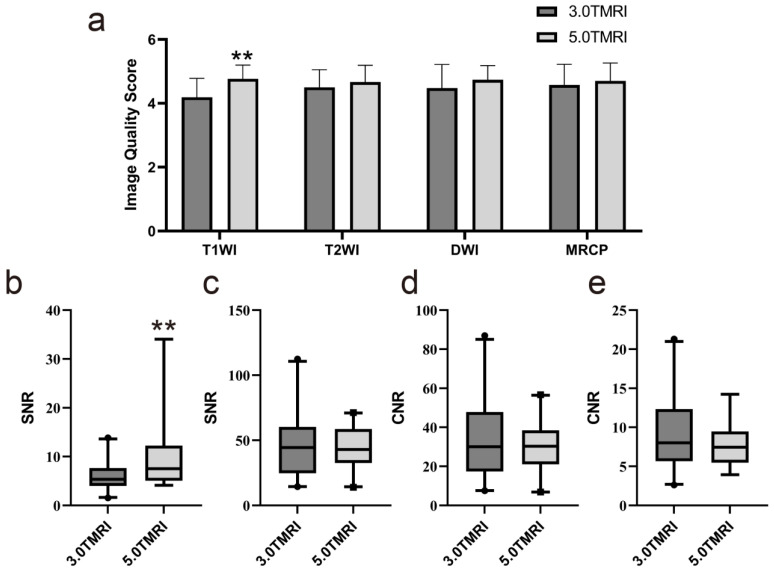
Subjective and objective image quality of 5.0 T MRI and 3.0 T. (**a**) Subjective image quality scores of 5.0 T and 3.0 T MRI. Signal-to-noises (SNRs) of lesions based on T1-weighted and T2-weighted sequences between 3.0 and 5.0 T MRI using the paired Wilcoxon tests are displayed in (**b**,**c**), respectively. Contrast–noise ratios (CNRs) were calculated and compared using the paired Wilcoxon tests on T1-weighted (**d**) and T2-weighted sequence (**e**) between 3.0 and 5.0 T MRI. CNR, contrast–noise ratio; SNR, signal-to-noise ratio; T1WI, T1-weighted imaging; T2WI, T2-weighted imaging; DWI, diffusion-weighted imaging; MRCP, magnetic resonance cholangiopancreatography. ** means *p* < 0.01.

**Figure 3:**
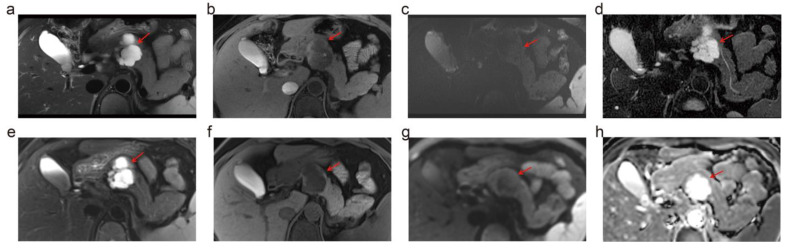
The images from 5.0 T and 3.0 T MRI of a 44-year-old female. This patient was suspected of serous cystadenoma, which was stable at imaging follow-up. 5.0 T MRI demonstrates a sharper contour of the lesion and more delicate details, including the thin septa within the lesion, the small central scar, and the relationship with the non-dilated main pancreatic duct, compared to the 3.0 T MRI. (**a**–**d**) show the T2-weighted imaging, T1-weighted imaging, DWI, and ADC map of 5.0 T MRI. (**e**–**h**) display the T2-weighted imaging, T1-weighted imaging, DWI, and ADC map of 3.0 T MRI. The red arrows refer to the lesion.

Figure 2a presents the subjective image quality assessments for MRI obtained at two different field strengths. Notably, T1WI at 5.0 T showed significantly higher image quality scores than at 3.0 T (*p* < 0.05). However, no significant differences were observed in the image quality scores of T2WI, DWI, and MRCP between the two field strengths (Figure 3). As for qualitative analysis, the SNR of T1WI was significantly higher at 5.0 T compared to 3.0 T (*p* = 0.008), but there is no significant difference in the SNR of T2WI between the two (Figure 2b–e). Moreover, there was no difference in CNR between 5.0 T and 3.0 T in both T1WI and T2WI (*p* = 0.269 and 0.269, respectively).

Supplementary Figure S2 illustrates shimming outcomes for the simulation model and a healthy male volunteer. Simulation results revealed that circularly polarized (CP) excitation without RF shimming led to magnetic field distribution non-uniformity. The application of RF shimming with the MLS algorithm enhanced uniformity in the upper abdominal region, decreasing the CV by 13.96%. Similar effects were also observed in the in vivo experiment, where RF shimming with the MLS algorithm reduced B1+ non-uniformity by 4.38% compared to CP excitation.

### 3.3. Depiction of PCL Imaging Features: 5.0 T vs. 3.0 T vs. MDCT

**Figure 4:**
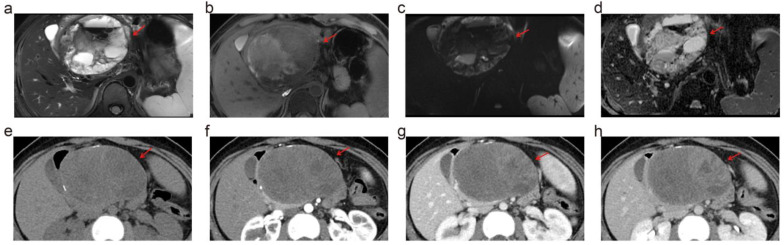
Solid pseudopapillary neoplasm in a 28-year-old woman. 5.0 T MRI ((**a**) for T2WI and (**b**) for T1WI) demonstrated the complex infrastructure of the tumor, including multiple septa and fluid–fluid levels within a portion of the lesion suggestive of intratumoral hemorrhage. The peripheral solid tumor component was hyperintense at DWI (**c**) and hypointense at the ADC map (**d**). These signs were highly suggestive of solid pseudopapillary neoplasm. On the contrary, peripheral eggshell-like calcification and a complex solid-cystic appearance with heterogeneous enhancement at multiphasic enhanced CT led to a misdiagnosis of mucinous neoplasm with malignant transformation at MDCT. (**e**–**h**) show the MDCT images in the plain scan, arterial phase, portal venous phase, and delayed phase. At pathologic analysis, this lesion was diagnosed as a solid pseudopapillary neoplasm. The red arrows refer to the lesion.

The maximum diameter of the largest cyst in the pancreas and the width of the MPD were measured, which revealed excellent consistency across 5.0 T MRI, 3.0 T MRI, and MDCT (cystic size: ICC = 0.947 and 0.976; the width of the MPD: 0.935 and 0.90; both are 3.0 T and MDCT compared with 5.0 T MRI, respectively). Moreover, in three cases, the main pancreatic duct was not clearly visible on MDCT due to peripancreatic fat infiltration (Figure 4).

**Figure 5:**
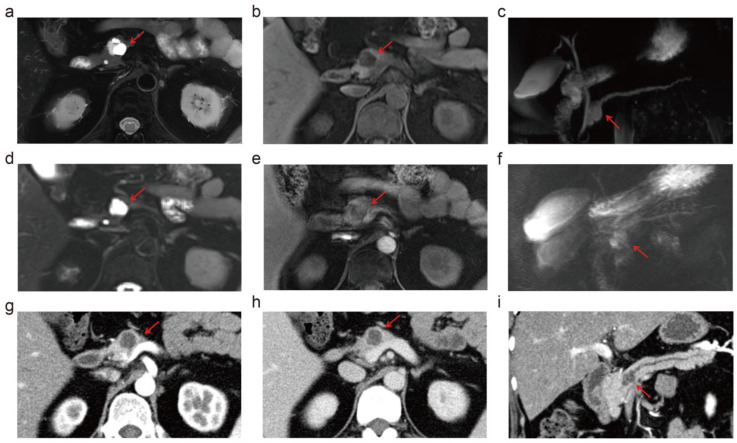
The images from 5.0 T MRI, 3.0 T MRI, and MDCT of a 56-year-old female. This patient was suspected of intraductal papillary mucinous neoplasm, which was stable at imaging follow-up. 5.0 T MRI demonstrates a sharper contour of the lesion and more delicate details, including the thin septa within the lesion and the communication between the cystic lesion and the mildly dilated main pancreatic duct (MPD), compared to the 3.0 T MRI. MDCT with curved planar reconstruction demonstrated the cyst–MPD communication as well but failed to show the thin septa within the lesion. (**a**–**c**) display the images from 5.0 T MRI of T2-weighted, T1-weighted, and MRCP, respectively. T2-weighted, T1-weighted, and MRCP images of 3.0 T MRI were attributed to (**d**–**f**), respectively. MDCT showed the lesions in arterial phase (**g**), portal venous phase (**h**), and curved planar reconstruction (**i**). The red arrows refer to the lesion.

For all patients, at least one cyst was detected at 5.0 T, 3.0 T MRI, and MDCT. 5.0 T detected more cysts in the same patients compared to 3.0 T and MDCT. More patients were detected with multiple cysts at 5.0 T MRI compared to 3.0 T MRI and MDCT (5.0 T vs. 3.0 T: *n* = 6 and 4 for all 21 patients; 5.0 T vs. CT: *n* = 11 and 8 for 33 patients) (Table 4). 5.0 T MRI found more patients with intracystic fluid and cystic lesions communicating with MPD than 3.0 T MRI. However, patients with mural nodules and cystic lesions with peripancreatic infiltration were more frequently detected on 3.0 T MR compared with 5.0 T MRI. 5.0 T MRI excelled over MDCT in assessing cyst features such as MPD dilation, intracystic septa, mural nodules, and cystic lesions communicating with MPD and intracystic fluid (Table 4). Both 5.0 T MRI and MDCT demonstrated three cases of peripancreatic infiltration (Figure 5).

### 3.4. Diagnostic Performance of PCL Characterization: 5.0 T MRI vs. 3.0 T MRI vs. MDCT

In the subset of patients who have undergone surgical resection (*n* = 15), we compared the diagnostic accuracy of 5.0 T MRI, 3.0 T MRI, and MDCT for categorizing PCLs against the pathological diagnoses. The categorization accuracy of 5.0 T MRI was notably high at 80%, compared to 40% for 3.0 T MRI and 36.6% for MDCT. Notably, 5.0 T MRI accurately identified lesion types in many cases that were misclassified by MDCT (Figure 4).

To assess the ability of each modality to determine the benign or malignant nature of PCLs, we compared the sensitivity and specificity across 5.0 T MRI, 3.0 T MRI, and MDCT based on pathological findings and patient follow-up data (Table 5). The sensitivity for 5.0 T MRI, 3.0 T MRI, and MDCT was 75% (9/12), 50% (3/6), and 27.3% (3/11), respectively. In terms of specificity, the respective values were 100% (23/23), 93.3% (14/15), and 86.4% (19/22), respectively. Moreover, in the accuracy of differentiating benign from malignant lesions, 5.0 T MRI demonstrated the highest accuracy (91.4%), followed by 3.0 T MRI (81%) and MDCT (66.7%).

## 4. Discussion

This study explored the feasibility of 5.0 T MRI for characterizing pancreatic cystic lesions (PCLs) compared to MDCT and 3.0 T MRI. Our findings reveal that, while the subjective and objective image quality of the 5.0 T and 3.0 T MRI were broadly equivalent, certain sequences (particularly T1WI) exhibit enhanced performance at 5.0 T. In terms of displaying lesion details, 5.0 T MRI was superior to CT, and it also exhibited modest advantages compared to 3.0 T MRI in certain aspects, such as cystic quantity and intracystic fluid. Crucially, when it comes to the specific categorization of cystic types and determining the benign or malignant nature of lesions, 5.0 T MRI demonstrates clear superiority over both CT and 3.0 T MRI. These findings suggest that 5.0 T MRI may be an effective tool in the precise diagnosis and management of PCLs.

Theoretically, an increase in magnetic resonance field strength corresponds to higher achievable image resolution and contrast, thereby leading to improvements in SNR and CNR, as well as clearer image details [17]. However, ultra-high field MRI also encounters various technical challenges, including the non-uniformity of the B0 field and B1 field, increased organ-specific absorption ratio value, accelerated attenuation rate of transverse magnetization, and black band artifacts caused by dielectric effects [18,19]. These challenges are especially pronounced in upper abdominal imaging. In our study, improved B1 field homogeneity was obtained by using an 8-channel parallel volumetric transmit coil, which automatedly tailored the transmit weightings for each subject and laid a good foundation for 5.0 T abdominal MRI. We further demonstrated the feasibility of a 5.0 T MRI for PCLs by comparing it with a 3.0 T MRI. Subjective image quality and SNR in T1WI of 5.0 T MRI were significantly higher than those in 3.0 T MRI. This finding aligns with the expectation that higher magnetic field strength contributes to improved SNR. T1WI is essential for diagnosing PCLs and pancreatic cancer, providing high-resolution images that enhance tissue contrast [20,21]. It allows for detailed characterization of pancreatic lesions, helping to differentiate between benign and malignant conditions. Additionally, T1WI is valuable for assessing necrosis, hemorrhage, and other critical features that affect clinical management and treatment decisions. Its role in early diagnosis can significantly influence patient outcomes and inform therapeutic strategies. However, this significant improvement was not observed in most other sequences of 5.0 T MRI. The reason may be attributed to the relatively small sample size, which may render it susceptible to the influence of abnormal values. Moreover, the relatively novel status of the 5.0 T technology may have limited the optimization of imaging sequences.

Moreover, 5.0 T MRI demonstrated comparable consistency with 3.0 T MRI and MDCT in measuring cyst size and MPD width, which means that 5.0 T MRI provides an equally clear and accurate depiction of the fundamental characteristics of the lesions as compared to conventional radiology. Notably, the superior clarity of 5.0 T MR enables it to detect more cystic lesions compared to the other two imaging modalities, which may be of significant benefit for patients with multiple cystic tumors. In the display of lesion details, 5.0 T MRI presents prominent advantages compared to MDCT and holds a comparable, if not superior, stance to 3.0 T MRI in certain aspects. The clarity of pancreatic duct visualization on 5.0 T images surpassed that on 3.0 T and MDCT images. Therefore, 5.0 T MRI exhibits the potential to compensate for the obscured visualization of the pancreatic duct resulting from peripancreatic fat infiltration. With future advancements and optimizations in sequences and imaging parameters, the capabilities of 5.0 T MRI in evaluating cystic lesions are expected to be further augmented.

Moreover, our study found that 5.0 T MRI yielded higher sensitivity, specificity, and accuracy in the differentiation of malignant lesions compared to MDCT and 3.0 T MRI. For the lesion categorization diagnosis, the diagnostic accuracy of 5.0 T MRI also surpassed those of MDCT and 3.0 T MRI. As is well known, PCL encompasses various lesions, each requiring distinct treatment approaches [22]. Given the potential complexities and risks of surgery, accurately identifying and assessing PCLs is crucial for optimal patient management [23]. Therefore, differentiating cystic lesions and assessing malignancy contributes to the accurate treatment and optimal management of PCLs. Our study included a comprehensive spectrum of PCLs, rendering it highly representative. Additionally, a subset of patients in our study underwent surgery, while others followed current clinical guidelines for regular follow-up. This aligns with the current standards for PCL treatment, reinforcing the reference value of our study. Our study demonstrates that 5.0 T MRI has a significant advantage over 3.0 T MRI and MDCT in terms of clinical diagnosis, which underscores the significant advantage of 5.0 T MRI over lower field strengths in aiding clinical decision-making. However, it is noteworthy that in cases where malignancy was misdiagnosed at 3.0 T MR and MDCT, three of which were also incorrectly assessed by 5.0 T MRI: one involving a rare cystic tumor (intraductal oncocytic papillary neoplasm) and the two others exhibiting focal high-grade dysplasia in IPMN. Their imaging signs were more subtle than invasive cancer, thus making detection more challenging. On the other hand, the dynamic contrast-enhanced sequence was not included in this preliminary study, which could be beneficial for characterizing pancreatic cystic lesions and detecting signs of malignancy. Therefore, future studies incorporating dynamic contrast-enhanced sequences for characterizing PCLs are warranted.

This study, while providing valuable insights, has several limitations. Firstly, this prospective study was carried out in a single center with a small size of enrolled patients, which may lead to statistical bias and selection bias. Moreover, in order to minimize the additional procedures for each patient while also considering the logistical constraints and patient willingness, a majority of 35 patients (*n* = 21) underwent both 5.0 T and 3.0 T MRI. Although the number of patients in this subset is sufficient for effective comparison, large-sample and multi-center studies are necessary to validate our findings. Secondly, in our malignancy assessments, lesions demonstrating malignant potential, low-grade malignancies, and invasive cancer were all categorized as malignant without further specific grading. The reason is that our study has a relatively small number of cases and a wide variety of diseases, and dividing all subjects into too many subcategories may compromise statistical power. Moreover, this classification method is consistent with clinical treatment [24]. Thirdly, the follow-up period was relatively short, which means that the absence of changes in lesions within this timeframe cannot conclusively indicate benign and may potentially lead to inaccuracies in our patient classification. In addition, the time interval between 5.0 T and 3.0 T MR scans could potentially influence the size of lesions, which might affect comparability. However, our follow-up results suggest that while there are differences in lesion detail, the consistency in lesion size between 5.0 T and 3.0 T MRI remains relatively high (ICC = 0.947). Finally, our analysis was limited to unenhanced scans using 5.0 T MRI, and the incorporation of contrast-enhanced scans could potentially enhance diagnostic efficacy and detail visualization.

## 5. Conclusions

In conclusion, 5.0 T MRI demonstrates significant potential in detecting PCLs, providing sufficient image quality. Moreover, 5.0 T MRI shows improved clinical diagnostic accuracy and differentiation of malignancy, which is crucial for making informed clinical decisions. These findings suggest the value of 5.0 T MRI in the diagnosis and management of PCLs, though its full potential may be further realized with technological advancements and more extensive studies.

## Figures and Tables

**Table 1:** MR imaging parameters.

Parameter		5.0 T (uMR Jupiter)	3.0 T (MAGNETOM Skyra )	3.0 T (GE Discovery MR 750)	3.0 T (Ingenia CX )
T1WI	TR * (ms)	~4.28	4.11	4.2	3.3
	TE (ms)	1.78	1.31–2.54	2.6	1.56
	FA (degrees)	10	12	15	12
	FOV	300 × 400	380 × 380	260 × 330	300 × 420
	Matrix (mm)	250 × 416	320 × 320	224 × 384	250 × 304
	Total acquisition time (s)	17	18	17	17
T2WI	TR (ms)	~5454	2390	2800	2000
	TE (ms)	83.2	81	90	80
	FA (degrees)	130	90	90	90
	FOV	300 × 380	400 × 400	340 × 340	380 × 380
	Matrix (mm)	204 × 304	256 × 256	224 × 320	304 × 304
	Total acquisition time (s)	260	250	275	240
DWI	TR (ms)	~5302	3000	4865	5000
	TE (ms)	64.3	53	70	63
	FA (degrees)	90	90	90	90
	FOV	120 × 280	400 × 400	400 × 400	300 × 380
	Matrix (mm)	123 × 288	128 × 128	128 × 128	128 × 128
	Total acquisition time (s)	397	210	160	270
MRCP	TR (ms)	~5454	2400	3045	1190
	TE (ms)	697.08	700	1300	600
	FA (degrees)	100	100	90	90
	FOV	352 × 352	384 × 384	320 × 340	260 × 360
	Matrix (mm)	264 × 352	256 × 256	224 × 384	260 × 240
	Total acquisition time (s)	238	205	210	245

* TR was influenced by the respiratory cycle. Abbreviations: TR, repetition time; TE, echo time; FA, flip angle; FOV, field of view; T1WI, T1-weighted imaging; T2WI, T2-weighted imaging; DWI, diffusion-weighted imaging; MRCP, magnetic resonance cholangiopancreatography.

**Table 2:** Patient demographic data.

Characteristic		Data
Age (y) *		57.11 (28–87)
Sex		
	Male	12 (34.29)
	Female	23 (65.71)
Diagnosis		
	IPMN	18 (51.43)
	MD-IPMN	0
	BD-IPMN	13 (37.14)
	MT-IPMN	5 (14.28)
	SCN	7 (20.00)
	MCN	4 (11.43)
	SPN	2 (5.71)
	PP	2 (5.71)
	IOPN	1 (2.86)
	pNET	1 (2.86)
Location		
	Head	17 (48.57)
	Body	4 (11.43)
	Tail	8 (22.86)
	Multiple sites	6 (17.14)
Treatment		
	Regular follow-up	20 (57.14)
	Pancreaticoduodenectomy	4 (11.43)
	Distal pancreatectomy	8 (22.86)
	Other	3 (8.57)

Note—Unless otherwise indicated, data are numbers of patients, and data in parentheses are percentages. * Data are the mean, and data in parentheses are the range. Abbreviations: IPMN, intraductal papillary mucinous neoplasm; MD-IPMN, main duct intraductal papillary mucinous neoplasm; BD-IPMN, branch duct intraductal papillary mucinous neoplasm; MT-IPMN, mixed-type intraductal papillary mucinous neoplasm; SCN, serous cystic neoplasm; MCN, mucinous cystic neoplasm; SPN, solid pseudopapillary neoplasm; PP, pancreatic pseudocyst; IOPN, intraductal oncocytic papillary neoplasm; pNET, pancreatic neuroendocrine tumor.

**Table 3:** Interobserver agreement of evaluating the image quality of 3.0 T and 5.0 T MRI.

			Observer 1	Observer 2	ICC	*p* *	*p* #
Image Quality Score	T1WI	3.0 T	4.14 ± 0.57	4.24 ± 0.62	0.596	0.002	<0.001
		5.0 T	4.73 ± 0.46	4.8 ± 0.41	0.825	<0.001	
	T2WI	3.0 T	4.48 ± 0.6	4.52 ± 0.51	0.603	0.001	0.16
		5.0 T	4.62 ± 0.59	4.71 ± 0.46	0.839	<0.001	
	DWI	3.0 T	4.52 ± 0.75	4.43 ± 0.75	0.919	<0.001	0.053
		5.0 T	4.76 ± 0.44	4.71 ± 0.46	0.882	<0.001	
	MRCP	3.0 T	4.54 ± 0.66	4.62 ± 0.65	0.910	<0.001	0.354
		5.0 T	4.65 ± 0.59	4.75 ± 0.55	0.854	<0.001	
Signal-to-Noise Ratio	T1WI	3.0 T	5.97 ± 2.99	6.35 ± 2.91	0.954	<0.001	0.008
		5.0 T	8.97 ± 5.09	8.42 ± 3.76	0.884	<0.001	
	T2WI	3.0 T	47.67 ± 27.35	48.27 ± 25.74	0.788	<0.001	0.552
		5.0 T	44.54 ± 17.08	47 ± 19.02	0.935	<0.001	
Contrast–Noise Ratio	T1WI	3.0 T	9.31 ± 4.69	9.42 ± 3.76	0.792	<0.001	0.269
		5.0 T	7.65 ± 2.89	6.08 ± 3.11	0.801	<0.001	
	T2WI	3.0 T	34.65 ± 21.97	35.37 ± 22.51	0.792	<0.001	0.269
		5.0 T	30.65 ± 14.31	32.82 ± 16.11	0.955	<0.001	

Note: Data are displayed as mean ± standard deviation. *p* * was from ICC analysis. *p* # was from paired Wilcoxon tests between 3.0 T and 5.0 T MRI. Abbreviations: T1WI, T1-weighted imaging; T2WI, T2-weighted imaging; DWI, diffusion-weighted imaging; MRCP, magnetic resonance cholangiopancreatography.

**Table 4:** Imaging findings of pancreatic cystic lesions in MDCT, 3.0 T MRI, and 5.0 T MRI.

Morphologic Finding		Number of Lesions (21 Patients)		Number of Lesions (33 Patients)	
		3.0 T MRI	5.0 T MRI	MDCT	5.0 T MRI
Location	Head	20	21	22	31
	Body	7	10	15	25
	Tail	5	9	19	26
	Total	32	40	56	82
Extent of MPD dilation					
	Head	0	1 (4.76)	0	1 (3.03)
	Body and tail	3 (14.29)	2 (9.52)	3 (9.09)	3 (9.09)
	Diffuse	7 (33.33)	7 (33.33)	4 (12.12)	9 (27.27)
Septa		15 (71.4)	15 (71.4)	15 (45.45)	23 (69.69)
Mural nodules		7 (33.33)	3 (14.29)	7 (21.21)	9 (27.27)
Communication with MPD		12 (57.14)	14 (66.66)	17 (51.51)	18 (54.55)
Peripancreatic infiltration		3 (14.29)	2 (9.52)	3 (9.09)	3 (9.09)
Intracystic fluid		1 (4.76)	3 (14.29)	1 (3.03)	7 (21.21)

Abbreviations: MPD, main pancreatic duct.

**Table 5:** Sensitivity, specificity, and accuracy of MDCT, 3.0 T, and 5.0 T MRI for the differentiation of benign from malignant lesions.

	Sensitivity (%)	Specificity (%)	Positive Predictive Value (%)	Negative Predictive Value (%)	Accuracy (%)
MDCT	27.3	86.4	50	70.4	66.7
3.0 T MRI	50	93.3	75	82.4	81
5.0 T MRI	75	100	100	88.5	91.4

Receiver operating characteristic (ROC) curve analysis was performed to evaluate the diagnostic performance of 5.0 T MRI, 3.0 T MRI, and MDCT to distinguish between benign and malignant lesions. The diagnostic performance of 5.0 T MRI was superior to that of both 3.0 T MRI and MDCT (5.0 T vs. 3.0 T MRI: AUC = 0.833 and 0.717, respectively; 5.0 T MRI vs. MDCT: AUC = 0.909 and 0.568, respectively).

## Data Availability

Data are contained within the article and Supplementary Materials.

## Supplementary Materials

The following supporting information can be downloaded at https://www.mdpi.com/article/10.3390/diagnostics14212457/s1, Figure S1: The example of region of interest (ROI) selection for the pancreatic cystic lesions at 5.0 T (a) and 3.0 T MRI (b).;Figure S2: Shimming outcomes for the simulation model and a healthy male volunteer.
