# Supplementary material for: Clinical Feasibility of 5.0 T MRI/MRCP in Characterizing Pancreatic Cystic Lesions: Comparison with 3.0 T and MDCT
Source: Diagnostics (Basel). 2024 Nov 2;14(21):2457. doi: 10.3390/diagnostics14212457 (PMC11545645; doi:10.3390/diagnostics14212457)
Supplement: Supplementary file 1 [file diagnostics-14-02457-s001.zip › diagnostics-3264585-supplementary.pdf]

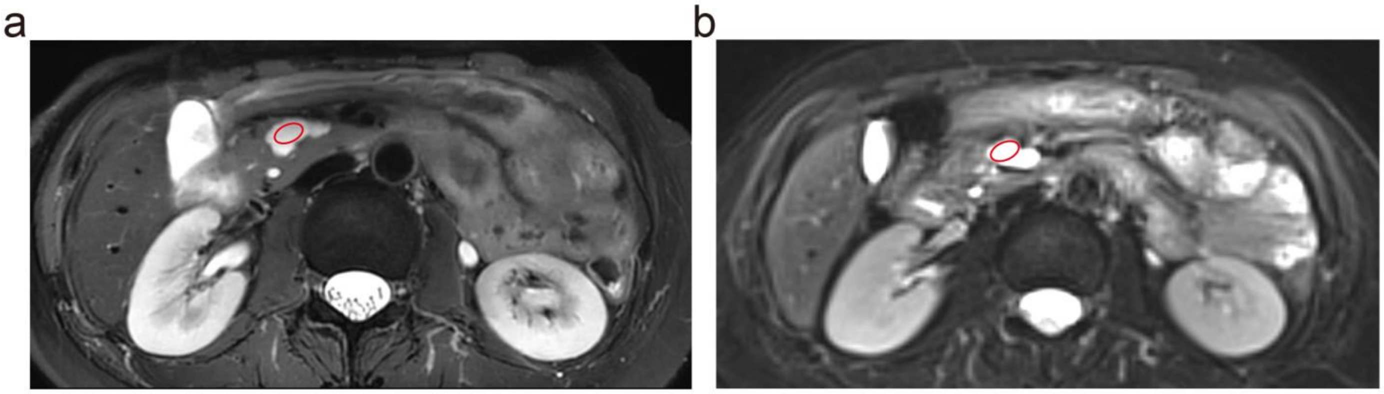

Figure S1. The example of region of interest (ROI) selection for the pancreatic cystic lesions at 5.0 T (**a**) and 3.0 T MRI (**b**).The red circles indicate the ROI.

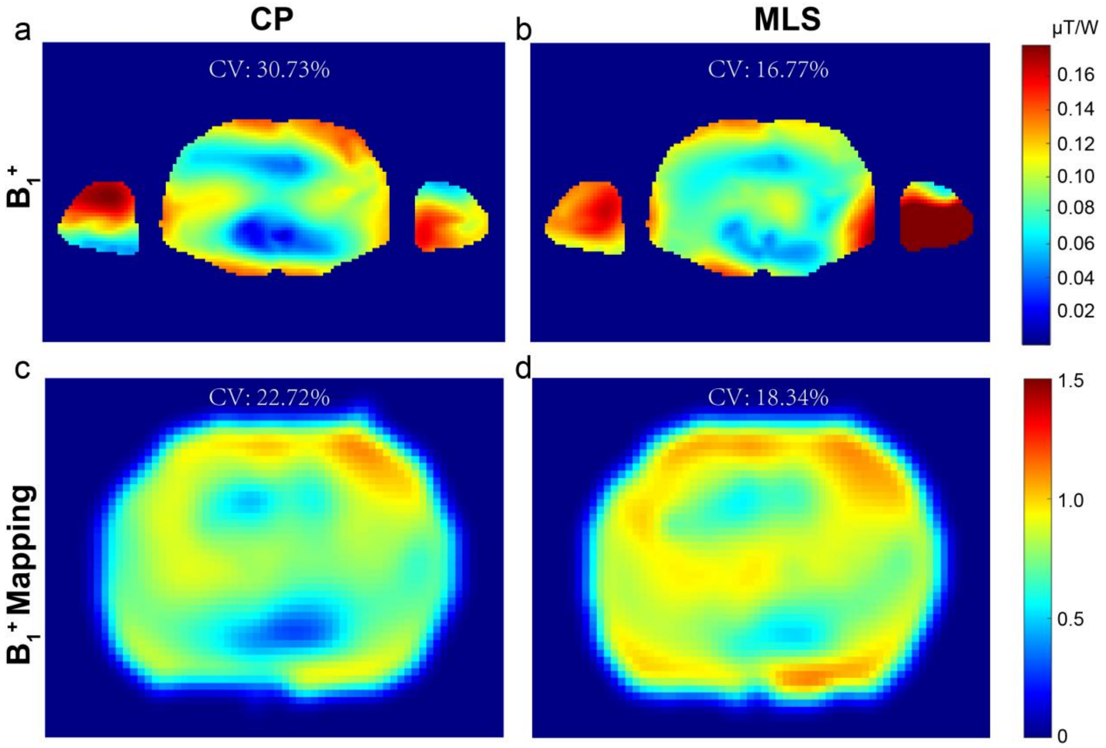

Figure S2. Shimming outcomes for the simulation model and a healthy male volunteer. (a) Simulation results showed that CP excitation without RF shimming led to a non-uniform magnetic field distribution (CV=30.73%); (b) RF shimming with the MLS algorithm in simulation improved field uniformity, decreasing the CV to 13.96%; (c) In-vivo, CP excitation without RF shimming resulted in a B1+ non-uniformity of 22.72%; (d) In vivo, RF shimming with the MLS algorithm further improved B1+ field uniformity, achieving a CV of 18.34%. CP, circularly polarized; MLS, magnitude least squares; CV, coefficient of variation.
